# Strategies to Reduce the Burden of Prostate Cancer and Their Acceptability in Africa: A Scoping Review Protocol

**DOI:** 10.3390/ijerph23020172

**Published:** 2026-01-30

**Authors:** Xolelwa Ntlongweni, Sibusiso Cyprian Nomatshila, Wezile Wilson Chitha, Nomfuneko Sithole, Sikhumbuzo Advisor Mabunda

**Affiliations:** 1 School of Public Health, Walter Sisulu University, Mthatha 5117, South Africa; snomatshila@wsu.ac.za (S.C.N.); wchitha@wsu.ac.za (W.W.C.); nosithole@wsu.ac.za (N.S.); smabunda@wsu.ac.za (S.A.M.); 2 Society and Health Research Institute, Walter Sisulu University, Mthatha 5117, South Africa; 3 Global Centre for Human Resources for Health Intelligence, Walter Sisulu University, East London 5247, South Africa; 4 Institute for Clinical Governance & Healthcare Administration, Walter Sisulu University, East London 5247, South Africa; 5 School of Population Health, University of New South Wales, Sydney 2052, Australia; 6 George Institute for Global Health, University of New South Wales, Sydney 2031, Australia

**Keywords:** prostate cancer, prevention, acceptability

## Abstract

**Highlights:**

**Public health relevance—How does this work relate to a public health issue?**

- Prostate cancer is one of the leading cancers affecting men in Africa, with high morbidity and mortality rates due to late detection and limited access to preventive services.
- Mapping prevention and early detection strategies and their acceptability addresses a critical public health need to reduce disease burden and improve population health outcomes.

**Public health significance—Why is this work of significance to public health?**

- Understanding which strategies are effective and culturally acceptable will inform targeted interventions that are more likely to be adopted in African contexts.
- The review will highlight gaps in knowledge and research, guiding future public health initiatives and resource allocation for prostate cancer prevention and control.

**Public health implications—What are the key implications or messages for practitioners, policy makers and/or researchers in public health?**

- Practitioners can use the findings to implement evidence-based, culturally sensitive prevention and early detection programs tailored to African populations.
- Policy makers and researchers can leverage the evidence to prioritize policies, funding, and research that enhance the acceptability and effectiveness of prostate cancer prevention strategies across African countries.

**Abstract:**

Background/Objectives: Prostate cancer is a major public health concern and a leading cause of cancer-related morbidity and mortality among men worldwide, with disproportionately high mortality rates in sub-Saharan Africa. Late diagnosis, limited access to screening services, low health-seeking behaviour, and sociocultural barriers contribute to poor outcomes, particularly in rural settings. However, evidence on effective and context-appropriate strategies for prostate cancer prevention and early detection in African countries remains fragmented. Objective: This scoping review protocol aims to map and synthesize existing evidence on strategies implemented to reduce the burden of prostate cancer in Africa, with a focus on prevention, screening, community engagement approaches, and their acceptability. Methods: This scoping review protocol will be conducted in accordance with established methodological frameworks and reported following the PRISMA-ScR guidelines. A comprehensive search will be undertaken in PubMed (MEDLINE), EBSCOhost, ScienceDirect, CINAHL, and ProQuest. Studies conducted in African countries and published in English, French, or Portuguese will be included. Study selection and data extraction will be managed using Covidence, and findings will be summarized descriptively and thematically. Expected Results: This review is expected to identify and categorize existing prostate cancer prevention and early detection strategies implemented across African settings. Gaps in evidence, differences in implementation, and reported levels of acceptability among men are anticipated to be highlighted. Conclusions: The completed scoping review is expected to provide a comprehensive overview of prostate cancer prevention and early detection strategies implemented in Africa and to identify gaps in evidence. The evidence will inform the development of culturally responsive and context-specific interventions, with particular relevance for rural South African settings. This manuscript presents a protocol for a scoping review; no review findings are reported.

## 1. Introduction

Prostate cancer is a malignant tumor that originates in the prostate gland, a small organ in the male reproductive system located below the bladder [1]. It is one of the most widely diagnosed cancers in men worldwide and is strongly age-related [2], with incidence increasing markedly after the age of 50 and peaking in older populations [2]. In 2020, prostate cancer was the second most frequently diagnosed cancer in men globally, accounting for more than 1.4 million new cases [2,3], reinforcing its significance as a major public health concern [4].

The development and progression of prostate cancer are influenced by a combination of biological, genetic, and lifestyle-related factors [5], including advanced age, family history, genetic susceptibility (such as BRCA mutations), hormonal influences, dietary patterns, and physical inactivity [5]. Emerging evidence suggests that lower testosterone levels at the time of diagnosis may be associated with a more aggressive disease phenotype [5]. Ethnicity also plays a significant role, shaped by interactions between genetic, biological, and social determinants of health. Men of African descent experience a higher risk of prostate cancer and are more likely to present with aggressive disease and poorer outcomes [6,7].

Globally, prostate cancer incidence and mortality rates show marked geographical differences. High-income countries such as those in North America and Europe report the highest incidence rates, a pattern largely attributed to widespread prostate-specific antigen (PSA) screening, early detection, and robust cancer surveillance systems [3,8], but relatively lower mortality due to timely diagnosis and improved access to effective treatment. In contrast, many low- and middle-income countries report lower incidence but disproportionately higher mortality [8,9], reflecting disparities in healthcare infrastructure, availability of screening, and access to diagnostic and treatment services.

These inequalities are particularly evident in Africa. Although reported incidence rates in sub-Saharan Africa remain lower than those in high-income regions, mortality rates are substantially higher [9,10], with mortality-to-income ratios often exceeding 0.5, compared with ratios below 0.2 in high-income settings [10]. This reflects poorer survival outcomes and is largely attributable to late-stage diagnosis, limited cancer registries, underreporting, and insufficient access to diagnostic and treatment services [8,11]. Variations between African countries are influenced by differences in cancer surveillance systems and screening practices rather than true differences in disease occurrence [12,13].

Beyond structural healthcare limitations, sociocultural factors and economic factors, such as low health literacy, stigma, financial constraints, and delayed health-seeking behaviour, further contribute to late presentation and poor outcomes [11]. Consequently, many men in African settings are diagnosed at advanced stages of the disease, when curative treatment options are limited or unavailable.

Despite the recognized importance of early detection and prevention, prostate cancer screening and prevention strategies remain highly contested in African settings. Unlike breast or cervical cancer, prostate cancer screening has not been uniformly adopted across African health systems, partly due to ongoing debates regarding the benefits and harms of prostate-specific antigen (PSA) testing, limited diagnostic follow-up capacity, and concerns about overdiagnosis and overtreatment [14,15,16]. In many African countries, the absence of organized screening programmes means that prevention efforts are often opportunistic, fragmented, and dependent on individual healthcare-seeking behaviour rather than system-level interventions [17]. These challenges highlight the need to better understand how prostate cancer prevention and early detection strategies are currently conceptualized, implemented, and experienced within African contexts.

In addition, community engagement and acceptability play a critical role in shaping the effectiveness of prostate cancer prevention initiatives. Evidence from African and other low- and middle-income settings indicates that men’s perceptions of masculinity, fear of cancer diagnosis, limited awareness, and mistrust of health systems significantly influence participation in screening and prevention programmes [18,19]. Community-based and culturally responsive approaches, including health education, involvement of community leaders, and integration into primary healthcare services, have been proposed as promising strategies to improve uptake and acceptability [20]. However, evidence on how such approaches have been applied, evaluated, and adapted across diverse African settings remains scattered and inconsistently reported.

Given the growing public health burden of prostate cancer in Africa, prevention strategies are critical to reducing morbidity and mortality. These encompass primary prevention aimed at reducing risk and health promotion, secondary prevention through early detection and screening, and tertiary prevention focused on disease management and prevention of complications [2]. While several African countries and regional bodies have developed prostate-cancer-related policies and guidelines [11,21], implementation remains fragmented due to resource constraints, workforce shortages, variable acceptability of interventions, and inadequate diagnostic infrastructure [22].

Despite these efforts, evidence on prostate cancer prevention and early detection strategies implemented and evaluated within African contexts remains fragmented. There is a need to systematically map existing strategies, assess their acceptability, and identify gaps in implementation. Therefore, this scoping review protocol aims to map and synthesize existing evidence on prostate cancer prevention strategies in Africa, with a particular focus on early detection, acceptability, and community-based approaches relevant to African and especially rural South African settings.

## 2. Materials and Methods

### 2.1. Study Design

The scoping review protocol is developed in accordance with the methodological framework proposed by Arksey and O’Malley and further refined by Levac et al. [23]. The planned review will follow six stages: (i) identifying the research questions; (ii) identifying relevant studies; (iii) selecting studies; (iv) charting the data; (v) collating, summarizing, and reporting the results; and (vi) optional consultation with relevant stakeholders.

The protocol is reported in accordance with the Preferred Reporting Items for Systematic Reviews and Meta-Analyses Extension for Scoping Reviews (PRISMA-ScR) guidelines. The protocol has been registered on the Open Science Framework (OSF) to promote transparency (registration ID: https://doi.org/10.17605/OSF.IO/Y5C3Q).

### 2.2. Eligibility Criteria

Eligibility criteria were developed using the Population-Concept-Context (PCC) framework, as recommended for scoping reviews.

- Population: Men in African countries
- Concept: Prostate cancer prevention and control strategies, including primordial, primary, secondary, and tertiary prevention; health promotion; early detection; management; rehabilitation; and palliation
- Context: Any healthcare, community, or policy setting within Africa

Inclusion Criteria

- Peer-reviewed primary studies conducted in African countries
- Studies reporting prostate cancer prevention or control strategies
- Quantitative, qualitative, and mixed-methods study designs
- Studies published in English, French, or Portuguese

Exclusion Criteria

- Studies conducted outside Africa
- Non-peer-reviewed literature (e.g., editorials, opinion pieces, commentaries)
- Animal studies

Review Questions

The scoping review will be guided by the following questions:

1. What prostate cancer prevention and control strategies have been implemented in Africa?
2. What enablers, including digital health approaches, support the delivery of these strategies?
3. What barriers hinder prostate cancer prevention and control in African settings?
4. How acceptable are prostate cancer prevention and control interventions to patients and communities

### 2.3. Information Sources and Search Strategy

A comprehensive and reproducible search strategy will be developed using a combination of controlled vocabulary and free text terms. The search will include the following key concepts: prostate cancer, prevention and control strategies, screening and surgical interventions, acceptability, and African settings. Search terms related to prostate cancer will include “prostate cancer”. Prevention and intervention concepts will include “prevention”, “strategies”, “screening”, and “orchidectomy”. Acceptability will be captured using the term “acceptability”. Geographic coverage will be ensured using “Africa” and “region” and country-specific terms, including “Africa” OR “East Africa” OR “West Africa” OR “Central Africa” OR “Southern Africa”, as well as individual country names (e.g., South Africa, Nigeria, Kenya, Ghana, Uganda, Ethiopia, Zimbabwe, among others).

*These concepts will be combined using Boolean operators as follows:*

“Prostate cancer” AND ((“prevention” OR “early detection” OR “health promotion” AND strategies) OR “orchidectomy” OR “surg*” “screen*”) AND “acceptability “AND (Africa* OR individual African country names) *.

Africa* OR Algeria OR Angola OR Benin OR Botswana OR Burkina Faso OR Burundi OR Cabo Verde OR Cameroon OR Central African Republic OR Chad OR Comoros OR Democratic Republic of the Congo OR Republic of Congo OR Cote d’Ivoire OR Ivory Coast OR Djibouti OR Egypt OR Equatorial Guinea OR Eritrea OR Eswatini OR Swaziland OR Ethiopia OR Gabon OR Gambia OR Ghana OR Guinea OR Guinea-Bissau OR Kenya OR Lesotho OR Liberia OR Libya OR Madagascar OR Malawi OR Mali OR Mauritania OR Mauritius OR Morocco OR Mozambique OR Namibia OR Ni-ger OR Nigeria OR Rwanda OR Sao Tome and Principe OR Senegal OR Seychelles OR Sierra Leone OR Somalia OR South Africa OR South Sudan OR Tanzania OR Togo OR Tunisia OR Uganda OR Zambia OR Zimbabwe OR Est Africa OR West Africa OR Central Africa OR Southern Africa.

1 AND ((2 AND 3) OR 4 OR 5 OR 6 OR 7) AND 8 AND 9.

The search strategy will be adapted as appropriate for each database. Searches will be conducted in PubMed, CINAHL, ScienceDirect, and ProQuest, and will include studies published up to 30 November 2025.

### 2.4. Study Selection

Following the search, all identified records will be imported into reference management software (Covidence), and duplicates will be removed. Titles and abstracts will be screened against the eligibility criteria, followed by full-text screening of potentially relevant articles. Screening will be conducted independently by at least two reviewers (X.N. and N.S.). Any conflicting resolutions will be mediated by a senior team member (S.A.M.) who will serve as a third reviewer. All decisions regarding inclusion or exclusion, along with the justifications, will be systematically recorded. The study selection process will be documented using a PRISMA-ScR flow diagram.

### 2.5. Data Charting and Synthesis

A standardized data charting form will be used to extract key study information, including publication year, country, study design, intervention characteristics, setting, and reported barriers, enablers, and acceptability. The data charting form may be refined iteratively during the review process. Extracted data will be summarized descriptively using narrative synthesis and tabular formats. In keeping with the objectives of a scoping review, the synthesis will focus on mapping the context, nature, and characteristics of prostate cancer prevention and control strategies in African contexts, rather than assessing intervention effectiveness.

### 2.6. Critical Appraisal

In line with scoping review methodology, no formal assessment of methodological quality or risk of bias is planned. As this manuscript presents a scoping review protocol, results will be reported in a subsequent publication following completion of the review.

## 3. Study Records

### 3.1. Data Management

Following the search process, selected articles will be transferred to EndNote X9 (Thomson Reuters, Philadelphia, PA, USA) for reference management and duplicate removal. De-duplicated records will then be uploaded to Covidence to facilitate screening and data extraction. Extracted data will be exported from Covidence into Microsoft Excel for data management, organization, and descriptive analysis. Study records and associated documents will be securely housed on a password-protected, server-based platform with access restricted to the review team.

#### 3.1.1. Selection of Sources of Evidence

Two independent reviewers (X.N. and N.S.) will oversee the study selection process. Titles and abstracts will first be screened against the eligibility criteria. Full-text articles will be retrieved for records deemed potentially relevant or where eligibility is unclear. Disagreements at any stage will be resolved through discussion, with a third reviewer (S.A.M.) acting as an arbitrator if consensus cannot be reached. Reasons for exclusion at full-text stage will be documented. The study selection process will be reported using a PRISMA-ScR flow diagram.

#### 3.1.2. Handling Ambiguities and Duplicate Reports

To address potential challenges in determining study eligibility, a set of guiding rules will be established. A frequent source of ambiguity arises when the study reports lack sufficient methodological detail. In such cases, the corresponding published protocol will be consulted to obtain additional information. Given limitations in time and resources, authors will not be contacted for clarification. Instead, such studies will be marked as excluded but noted as “potentially applicable”. Another common issue involves duplicate publications from a single study. When multiple reports are identified, the versions containing the most comprehensive information will be retained, with all related documents appropriately cited.

### 3.2. Data Collection Process

After identifying the studies eligible for inclusion, relevant information will be gathered using a standardized data extraction template. All selected studies will undergo data extraction in Covidence. To ensure clarity and consistency, the form will first be tested and refined by the two reviewers using a sample of three randomly chosen studies that meet the inclusion criteria. Thereafter, each reviewer will independently extract data from the remaining articles to maintain objectivity and reduce bias.

#### Data Items Data Extraction Framework

Data will be charted systematically from all included studies using a standardized data charting form developed for this review. Extracted information will include (i) study identification details (author, year, country, and publication type; (ii) study characteristics (aims, design, and setting); (iii) participant characteristics; (iv) a description of prostate cancer prevention or control strategies; and (v) reported outcomes related to implementation, barriers, enablers, and acceptability.

### 3.3. Data Synthesis and Analysis

Studies that meet the predefined inclusion criteria will be incorporated into the data synthesis. Findings will be summarized through a descriptive narrative, complemented by tables and visual representations where relevant. Where applicable, subgroup analyses may be conducted to explore variations in acceptability, barriers, and enablers influencing the implementation of interventions.

### 3.4. Future Research Directions and Possible Applications

The scoping review described in this protocol is intended to inform subsequent research activities once it has been completed. Specifically, the planned review will provide an evidence base to guide the identification of priority research gaps in prostate cancer prevention and early detection within African contexts. These identified gaps will be used to inform the design of future empirical studies, including planned quantitative and qualitative studies in a rural South African setting, focusing on the burden and distribution of prostate cancer, experiences of prostate cancer survivors, and knowledge, perceptions, and preventive practices among men in the general population.

## 4. Conclusions

This manuscript presents a protocol for a scoping review that aims to systematically map the existing evidence on prostate cancer prevention and control strategies in Africa. The manuscript outlines a transparent and methodologically rigorous approach that will provide the foundation for identifying the range of interventions implemented across diverse African contexts, as well as key barriers and enablers influencing their delivery and acceptability. The planned scoping review will generate a comprehensive evidence map to inform future research, policy development, and contextually appropriate prostate cancer prevention initiatives in African settings. Following publication, this protocol is intended for immediate implementation, with database searches to be conducted within two months of acceptance and updated, if necessary, to ensure the currency and relevance of the evidence prior to study selection and data charting.

## Data Availability

Data sharing does not apply to this protocol, as it does not involve the generation or analysis of datasets. Upon completion of the scoping review, any relevant data or materials will be made available in accordance with the ethical approvals and data sharing policies of the Walter Sisulu University Ethics Committee.

## Abbreviations

The following abbreviations are used in this manuscript:

PSA	Prostate-specific antigen
MIR	Mortality-to-incidence ratio
WHO	World Health Organisation
PICO	Population, Intervention, Control, Outcomes
RCT	Randomised control trial
PRISMA	Preferred Reporting Items for Systematic Review and Meta-Analysis
GRADE	Grading of Recommendations, Assessment, Development, and Evaluation
SAMRC	South African Medical Research Council
